# Oral Care Practice, Perception, and Attitude of Nurses in Intensive Care Units in Korea: A Questionnaire Survey

**DOI:** 10.3390/healthcare10102033

**Published:** 2022-10-14

**Authors:** Mi-Kyoung Jun

**Affiliations:** Sae·e Dental Clinic, 109-8, Songwon-ro, Jangan-gu, Suwon-si 16294, Gyeonggi-do, Korea; mijjomg@naver.com; Tel.: +82-10-4075-2116

**Keywords:** intensive care unit, nurses, oral care, oral health, oral hygiene, patient

## Abstract

Background: This cross-sectional study investigated the factors influencing oral care in patients in intensive care units (ICUs) by identifying the current oral care practice status, perceptions, and attitudes of ICU nurses in Korea. Methods: This study surveyed 240 nurses with work experience in the ICU using a self-report questionnaire consisting of 22 items on the status, perception, and attitude towards oral care in the ICU. Results: A total of 227 questionnaires were analyzed. The average age of the participants was 29.79 ± 5.92 years, and the medical ICU was the most type of ICU. The factors affecting the attitude towards oral care and its importance in ICU patients were the experience of working as a nurse (r = 0.336, *p* < 0.01), ICU work experience (r = 0.218, *p* < 0.01), and oral care practice frequency (r = 0.167 *p* < 0.05); these were found to be statistically significant. Conclusions: In this study, the interest of the nurses in oral care practice and education was high, but professional education and the regular implementation of oral care practices were insufficient. To address these problems, it will be necessary to improve oral care practices through dental intervention and education about oral care methods focused on ICU patients.

## 1. Introduction

Oral care is a basic nursing practice and intensive care unit (ICU) nurses can influence the oral health outcomes of their patients [1,2]. Oral care for patients in ICUs is the responsibility of the nurse. However, it has a lower priority as compared with other medical needs of the patient. Given the critical nature of the patient’s medical condition in ICUs, it is also difficult to continuously provide appropriate oral care [3,4].

Most ICU patients have a weakened immune system and are highly susceptible to oral infections [5]. They may be exposed to risks such as dry mouth caused by various drugs and oral candidiasis caused by the extensive use of antibiotics [6]. In addition, it is known that the main causes of ventilator-associated pneumonia (VAP), an infectious complication that occurs in ICU patients who have undergone endotracheal intubation, are the aspiration of bacteria from the pharynx and leakage of contaminated secretions around the endotracheal tube [7]. However, although it is important to provide oral care to prevent the colonization of bacteria that cause VAP, it is difficult to evaluate the oral condition of patients while they are intubated because access to the oral cavity during oral care is limited and there is a risk of changing the position or accidental extubation [8].

In several recent studies, various oral hygiene management methods such as the application of chlorhexidine, mechanical cleaning using a manual toothbrush, and administering 10% povidone-iodine to ICU patients, were the most common methods of oral care to prevent pneumonia [9,10]. The use of chlorhexidine for oral care of intubated patients significantly reduced the incidence of VAP, a major cause of mortality in ICU patients [11,12]. Therefore, it is important to emphasize oral care to prevent the growth of bacteria in the oral cavity through good oral hygiene management and to prevent deterioration of oral health and the onset of respiratory infections [13,14].

Although assessment and maintenance of oral health is an important task for nurses [15], there is insufficient evidence-based knowledge of methods to provide effective oral care [3,16]. Additionally, many studies indicate that there is lack of consensus on the evaluation methods of oral health conditions in ICU patients, the types of the products used in oral hygiene management and the frequency with which oral care was provided [15,17]. Although oral care protocols are in place, standardized evidence-based or practice-based guidelines in the ICU environment are often not followed, therefore there is a gap between the guidelines and their actual clinical application [18].

The quality of oral care provided by ICU nurses is influenced by the nurses’ perceptions of the importance of oral care and the professional educational programs provided to them [19]. However, to date, there are insufficient reports on the status, perception, and attitude of oral care practiced by nurses.

Therefore, the purpose of this study was to investigate the practice, perceptions, and attitude toward the oral care practices of nurses with experience in intensive care in Korea, and to analyze factors related to oral care to provide the basis for the preparation of evidence-based guidelines for the oral care of ICU patients.

## 2. Materials and Methods

### 2.1. Participants and Data Collection Procedures

This study conducted an online survey targeting 240 nurses with more than 6 months of work experience in the ICUs of 10 general hospitals in Korea. All study participants were informed on the purpose of the study, anonymity, and confidentiality, and consent was confirmed before they filled out the questionnaire. This study was conducted with the approval of the Institutional Review Board of Ajou University Hospital (No: AJIRB-SBR-SUR-21-245). Excluding 13 questionnaires with incomplete responses, the responses of 227 nurses were used for the final analysis.

### 2.2. Study Design

This was a cross-sectional study.

### 2.3. Survey Methods

For the structured questionnaire, a total of 22 questions were developed that modified the questions of the research tools used in previous studies [20,21] and supplemented the questionnaires on oral care practice and education by dental professionals. The questionnaire was reviewed by an expert panel consisting of 2 nurses from an ICU, 3 dental hospital professors, and 2 dental hygiene professors to obtain feedback on the content and effectiveness of the questions. Subsequently, a preliminary test was conducted on 15 nurses working in the ICUs of three general hospitals. Subsequently, the expert panel came to a consensus on all the items and finalized the questionnaire as follows:

General characteristics of participants: These were covered by a total of 7 questions that included queries on age, gender, educational background (3 years of college, bachelor’s degree in nursing, master’s degree in nursing, doctoral degree in nursing), position, total clinical and ICU work experience, and the type of ICU where the nurses were currently employed.

Actual oral care practice: Questions covered 6 areas including oral health status examination method, oral care products, oral care implementation time per day, oral health status evaluation period, and oral care practice frequency during nursing work over the past 3 months.

The perception attitude towards oral care practice and education: Questions comprised 8 items that sought to investigate whether oral care education was completed, whether oral care education was necessary, application of oral care practice guidelines, and perception of oral care practice.

The attitude towards the importance of oral care in nursing practice was recorded using a 5-point scale (ranging from very important to not very important).

### 2.4. Statistical Analysis

Statistical analysis was performed using Predictive Analytics Software (PASW), version 18.0 for Windows (SPSS Inc, Chicago, IL, USA). A normality test using the Shapiro–Wilk test resulted in a normal distribution. The general characteristics of nurses, their practice, perception, and attitude towards oral care were analyzed using descriptive statistics. Differences in oral care practice, perception, and attitude according to the general characteristics of nurses were analyzed by an independent-sample t-test. Pearson correlation analysis was conducted on the correlation between the nurse’s overall work experience, ICU work experience, oral care performance frequency, and attitude towards the need for oral care. All statistical analyses were analyzed at a significance level of 0.05.

## 3. Results

### 3.1. Participant Characteristics

As shown in Table 1, the average age of the participants was 29.79 ± 5.92 years, and females accounted for 70.9%. The level of nursing education that showed the highest frequency was a bachelor’s degree in nursing at 81.9%. Additionally, 31.7% and 35.2% of the subjects had nursing and ICU work experience of 2–4 years.

### 3.2. Oral Care Practice Status of Intensive Care Unit Nurses

‘Visual inspection’ and ‘palpation’ were the most used methods of evaluating oral health status. ‘After dinner’ was the most common time of the day for assessment and scored the highest at 26.9%. Of the patients who were unable to manage self-oral hygiene, the oral health status of 96% was evaluated daily. ‘Chlorhexidine’ accounted for 41.6% of the oral care products used, and ‘gauze’ for 25.6%. Over the 3 months before the survey, 52% of the surveyed nurses said that the frequency of oral care given to the patients in their charge was ‘daily’ (Table 2).

### 3.3. ICU Nurses’ Perception, and Attitudes toward Oral Care

ICU nurses’ awareness and attitudes towards oral care are presented in Table 3. It was found that 58.6% of the participants had never received oral care education, and 30.9% had their most recent oral care education in the previous 5 years. Regarding the need for oral care education, 82.8% answered they felt the ‘need’, and ‘oral hygiene management method’ was the highest with 21.5% as the item for which education was deemed necessary. More than half of the respondents did not know about the application of the oral care practice guidelines, and only 30.8% answered ‘yes’. As a result of identifying the frequency of oral care practice according to the guidelines based on the subjects who answered ‘yes’, 61.4% responded that it was applied to more than 70% of the patients in their charge.

Regarding the perception of whether the current frequency of oral care was sufficient to maintain oral care, 70% answered ‘sufficient’ and 30% answered ‘insufficient’. Furthermore, among the reasons why they responded ‘insufficient’, ‘too much work and not enough time’ was the highest at 41.4%.

The attitude towards the importance of oral care elicited responses that it was ‘important’ and ‘very important’ from 42.3% and 11.0% of the subjects, respectively, indicating that they felt that the importance of oral care was high.

### 3.4. Differences in Attitudes about the Importance of Oral Care According to the General Characteristics of ICU Nurses

Compared with staff nurses, the average score of the charge nurses was 3.81, indicating a more positive attitude towards the importance of oral care. In addition, according to the ICU work experience, the average score was 3.74 for more than 6 years, which was higher than the score of 3.48 for less than 5 years, and there was a statistically significant difference. (Table 4)

### 3.5. Differences in Attitudes towards the Importance of Oral Care According to Oral Care Perception and Educational Experience

The attitude towards the importance of oral care appeared to be high when the current oral care practice was ‘sufficient in maintaining oral care’ of the patient. It was found that the “existence oral care education experience” was statistically significantly higher. (Table 5)

### 3.6. Factors Related to Attitudes towards the Importance of Oral Care

The attitude towards the importance of oral care was significantly correlated with nursing clinical experience (r = 0.336, *p* < 0.01), ICU work experience (r = 0.218, *p* < 0.01), and oral care performance frequency (r = 0.167 *p* < 0.05) (Table 6).

## 4. Discussion

Our study identified differences in the educational experiences and perceptions and attitudes towards oral care among nurses in the ICUs. It also examined the variation in oral health status evaluation and oral care by nurses caring for ICU patients using different methods at varying frequencies. When evaluating the oral health status of ICU patients, it was confirmed that ‘visual inspection’ and ‘palpation’ were the most commonly used oral examination methods by nurses, making it a subjective evaluation. A direct comparison of the oral health status of ICU patients is possible only when a standardized and objective oral examination method is applied. However, the oral health status evaluation performed by many nurses relies on subjective evaluation unlike the evaluation of other medical signs and symptoms [18,22]. A subjective evaluation of oral health cannot differentiate between the common and highly prevalent oral diseases, namely dental caries and periodontal disease, from the impact of the ICU status on the oral health of these patients. This suggests that the involvement and cooperation of dental professionals is necessary for better oral care management in ICUs.

For oral care practice, responses to queries on the timing of the care given indicated that 26.9% and 22.2% of the care was given ‘after dinner’ and ‘before going to sleep’, respectively. The responses showed that 41.6% of the nurses applied the oral care product chlorhexidine at this time. In actual clinical practice, ideally, oral care should be given three times a day, but the questionnaire responses showed that the actual frequency was lower [4]. Additionally, many respondents used chlorhexidine as the product of choice for administering oral care. In oral hygiene management, toothbrushes, which are known to be effective in reducing bacteria in oral biofilms in the oral cavity, were found to be less commonly used than gauze. This was consistent with previous studies in which toothbrushes were not used due to the difficulty of brushing the teeth of intubated patients [3]. As for the oral health status evaluation frequency of patients who could and could not manage oral hygiene themselves, 70% of the responses were ‘everyday’, but the frequency of oral care practice for the nurses for the past 3 months was lower as reflected in the response “always” accounting for only 52% of nurses surveyed [4]. This difference is thought to be influenced by differences in the ICU environment or essential nursing tasks depending on the hospital.

Evidence-based knowledge is essential for the application of individualized oral hygiene management methods according to the oral health status of the patient in ICUs, subsequent to appropriate evaluation. Nevertheless, several earlier studies have shown that the knowledge of nurses in the field of oral care is insufficient, which is consistent with the result that 58.6% of the participants in this study were not provided with relevant oral care education [23,24]. Even those who received such education had completed the courses more than 5 years previously. Thus, there was a need to provide updated oral care training.

For the question regarding the necessity of providing oral care education, 82.8% of the respondents felt it was ‘needed’. The responses of 21% of the nurses indicated that the type of oral care education they would like to receive involved ‘oral hygiene management methods’. This supports earlier studies that reported that there was inadequate information available on the frequency and methods of oral care employed by the nurses [25,26]. As for the clinical application of the oral care practice guidelines, 69.2% answered ‘none’ implying that they did not give care as per the guidelines, and the frequency of performing the oral care practice guidelines was also low as indicated by the fact that only 24.3% responded ‘always’ to queries on how often they administered such care. This is probably because oral care was not given adequate importance not only in the nursing college courses but also in clinical practice [27].

Regarding the frequency of oral care practice, 70% of respondents felt that the current oral care performance was ‘sufficient’, and 30% of the respondents said that it was ‘insufficient’ due to the burden of many nursing tasks to be completed. This contrasted with a previous study [26], but among the reasons for the response of ‘insufficient’, it was confirmed that the lack of time and staff due to other nursing tasks were the main factors.

The attitude towards the importance of oral care was different from previous studies where 53.3% of the respondents answered ‘important’ or higher. Thus, the respondents in this study showed a negative attitude compared with the previous studies, which could be due to the difference in the organizational climate or ICU environment [24,25]. The attitude of the ‘charge nurses’ towards the importance of oral care was significantly higher statistically than that of ‘staff nurses’. This positive attitude of the charge nurse was reflected in most studies. This could be because the charge nurse was normally the person in charge of oral care education in actual clinical practice. In addition, the attitude towards the importance of oral care was higher when the ICU work experience of the nurses was ‘6 years or more’. A previous study also reported that a higher ICU work experience translated into greater knowledge about oral care clinical guidelines for VAP prevention [26,28]. These results suggest that the accumulation of clinical experience and knowledge resulting from the ICU work experience of nurses could result in an improved attitude towards oral care. Oral care perception and oral care education were related to the importance of oral care. Subjects who responded that the oral care practice was ‘sufficient’ and the oral care education experience was ‘yes’ showed a more positive attitude towards oral care. Therefore, it is necessary to prepare an oral care program and provide professional training for nurses to care for ICU patients. This would encourage the nurses to cultivate a positive attitude towards oral care. A plan must thus be prepared for ICU nurses with fewer years of experience seeking to enhance their knowledge of the importance of oral care.

The factors related to the attitude of the respondents towards the importance of oral care showing a statistically significant positive correlation were nursing clinical experience (r = 0.336, *p* < 0.01), ICU work experience (r = 0.218, *p* < 0.01), and oral care practice frequency (r = 0.167 *p* < 0.05). As described above, ICU nurses showed a positive attitude towards the importance of oral care, but it was found that they did not provide adequate care. Routine oral care is important because it can influence the mortality rate of ICU patients [11,12]. A change in the perception of nurses and institutional improvement is required so that oral care can become an essential part of nursing jobs.

This study was a cross-sectional survey using a structured questionnaire to evaluate the oral health status of ICU patients and to investigate the practice status, perception, and attitudes of Korean nurses related to oral care in ICUs. The study has a limitation in that it is difficult to generalize the research results due to differences in the practice of oral care guidelines between hospitals. Future studies will need to confirm the level of knowledge of ICU nurses about oral diseases and an understanding of oral care guidelines.

## 5. Conclusions

This study showed that Korean nurses had different consistency in oral health status evaluations and the oral care of ICU patients, and that they had not received sufficient training. However, they had a positive attitude towards the practice of oral care. Therefore, education of nurses on sustainable and appropriate oral care for ICU patients, and the development of a standardized and evidence-based oral care protocol are required.

## Figures and Tables

**Table 1:** General characteristics of participants (n = 227).

Variable	Mean ± SD	N	%
Gender			
Male		66	29.1
Female		161	70.9
Age(years)	29.79 ± 5.92		
Level of nursing education			
Three years of college		5	2.2
Bachelor’s degree		186	81.9
Master’s degree		34	15.0
Doctoral degree		2	0.9
Position			
Staff nurse		171	75.3
≥Charge nurse		56	24.7
Type of ICU			
Medical		65	28.6
Surgical		55	24.2
Neurological		21	9.3
Cardiac		8	3.5
Emergency		32	14.1
General		46	20.3
Experience working as a nurse (years)			
<2		35	15.4
2–4		72	31.7
5–7		63	27.8
8–10		17	7.5
≥10		40	17.6
Experience working in ICU (years)			
<2		48	21.1
2–4		80	35.2
5–7		53	23.3
8–10		16	7.0
≥10		30	13.2

SD, standard deviation. Abbreviation: ICU, intensive care unit.

**Table 2:** ICU nurses’ practice of oral care.

Variable	N	%
Oral examination method *		
History taking	76	9.6
Visual inspection: color of gums and tongue	196	24.7
Visual inspection: color of teeth	153	19.3
Palpation: tooth mobility	195	24.6
Palpation: pain in the patient	78	9.8
Olfactory test: foul odor	96	12.1
Time of day for oral care *		
before breakfast	66	14.0
after breakfast	80	16.9
after lunch	94	19.9
after dinner	127	26.9
just before bed	105	22.2
Oral care products used with patients *		
manual toothbrush	100	20.1
dental floss	27	5.4
mouthwash	25	5.0
chlorhexidine	207	41.6
oral cleaning solution (povidone)	7	1.4
toothpick	1	0.2
electric toothbrush	1	0.2
gauze	127	25.6
mouth care stick	2	0.4
Period of evaluation of oral health status of patients who cannot undertake self-oral hygiene		
do not check separately	1	0.4
on the day of admission	6	2.6
everyday	218	96.0
2–3 days	1	0.4
when the patient complains	1	0.4
Period of evaluation of oral health status of patients who can perform self-oral hygiene		
do not check separately	13	5.7
on the day of admission	34	15.0
everyday	161	70.9
2–3 days	7	3.1
when the patient complains	11	5.2
Frequency of oral care practice in the past 3 months		
none (within 10%)	1	0.4
rarely (20–30%)	6	2.6
sometimes (40–60%)	17	7.5
often (70–90%)	85	37.4
always (100%)	118	52.0

* Multiple responses possible.

**Table 3:** ICU nurses’ awareness, attitudes towards oral care.

Variable	N	%
Experience in oral care education		
No	133	58.6
Yes ^a^	94	41.4
The most recent oral care education period ^b^		
Within 6 months	21	22.3
Within 1 year	14	14.9
Within 5 years	29	30.9
Within 10 years	13	13.8
In college student	17	18.1
Application of oral care practice guidelines		
No	157	69.2
Yes	70	30.8
Frequency of application of oral care practice guidelines *		
none (within 10%)	5	7.1
rarely (20–30%)	8	11.4
sometimes (40–60%)	14	20.0
often (70–90%)	26	37.1
always (100%)	17	24.3
Perception of the need for oral care education		
not needed	39	17.2
Needed ^a^	188	82.8
Items for which oral care education was required ^b^*		
Tooth condition evaluation	137	19.7
Gum condition evaluation	128	18.4
Tongue condition evaluation	124	17.8
Oral hygiene management method	150	21.5
Dietary prescription	61	8.8
Criteria for dental referral	97	13.9
Perception of the frequency of oral care practice		
Sufficient	159	70.0
Insufficient ^a^	68	30.0
Reasons for Insufficiency ^b^*		
Does not know how to use the oral care products	6	5.2
Lack of oral care products	16	13.8
Lack of prior knowledge about oral care	16	13.8
Does not think oral care is important	5	4.3
A lot of nursing practice	48	41.4
No doctor’s prescription	9	7.8
Colleagues not performing oral care	5	4.3
No guidance provided by the nursing department	11	9.5
Attitudes towards the importance of oral care		
Very unimportant	2	0.9
Unimportant	14	6.2
Neutral	90	39.6
Important	96	42.3
Very important	25	11.0

^a^ Asked to answer sub-questions. ^b^ Incorrect answers. * Multiple responses possible.

**Table 4:** Differences in attitudes about the importance of oral care according to the general characteristics of ICU nurses.

Variable	Division	N	Mean ± SD	*p*
Gender	Male	66	3.44 ± 0.84	0.135
	Female	161	3.61 ± 0.78	
Position	Staff nurse	171	3.46 ± 0.76	0.001
	≥Charge nurse	56	3.81 ± 0.85	
Experience working in ICU (years)	≤5	155	3.48 ± 0.78	0.027
	≥5	72	3.74 ± 0.82	
Type of ICU	Medical	94	3.67 ± 0.79	0.082
	Surgical	87	3.41 ± 0.81	
	General	46	3.63 ± 0.77	

By independent *t*-test.

**Table 5:** Differences in attitudes towards the importance of oral care according to oral care perception and educational experience.

Variable	Division	N	Mean ± SD	*p*
oral care perception	Sufficient	159	3.64 ± 0.78	0.040
	Insufficient	68	3.40 ± 0.85	
oral care education experience	No	133	3.43 ± 0.80	0.001
	Yes	94	3.76 ± 0.77	

By independent *t*-test.

**Table 6:** Correlation between nursing clinical experience, ICU experience, frequency of oral care practice, and attitude towards the importance of oral care.

	Nursing Clinical Experience	ICU Experience	Frequency of Oral Care Practice
Attitude towards the importance of oral care	0.336 **	0.218 **	0.167 *

* *p* < 0.05, ** *p* < 0.01 by Pearson’s correlation analysis.

## Data Availability

Not applicable.

## References

[1] Ames N.J., Sulima P., Yates J.M., McCullagh L., Gollins S.L., Soeken K., Wallen G.R. (2011). Effects of systematic oral care in critically ill patients: A multicenter study. Am. J. Crit. Care.

[2] Munro C.L., Grap M.J. (2004). Oral health and care in the intensive care unit: State of the science. Am. J. Criti. Care.

[3] McNeill H.E. (2000). Biting back at poor oral hygiene. Intensive Crit. Care Nurs..

[4] Grap M.J., Munro C.L., Ashtiani B., Bryant S. (2003). Oral care interventions in critical care: Frequency and documentation. Am. J. Criti. Care.

[5] Moretti A., Flaitz D., Peninger M., Rex J. (2002). Evaluation of oral soft tissue lesions in ventilated patients. Oral Surg. Oral Med. Oral Pathol. Oral Radiol Endod..

[6] Jones H., Newton J., Bower E. (2004). A survey of the oral care practices of intensive care nurses. Intensive Crit. Care Nurs..

[7] Trouillet J.-L., Chastre J., Vuagnat A., Joly-Guillou M.-L., Combaux D., Dombret M.-C., Gibert C. (1998). Ventilator-associated pneumonia caused by potentially drug-resistant bacteria. Am. J. Respir. Crit. Care Med..

[8] Abidia R.F. (2007). Oral care in the intensive care unit: A review. J. Contemp. Dent. Pract..

[9] Liu C., Cao Y., Lin J., Ng L., Needleman I., Walsh T., Li C. (2018). Oral care measures for preventing nursing home-acquired pneumonia. Cochrane Database Syst. Rev..

[10] Gershonovitch R., Yarom N., Findler M. (2020). Preventing Ventilator-Associated Pneumonia in Intensive Care Unit by improved Oral Care: A Review of Randomized Control Trials. SN Compr. Clin. Med..

[11] Fourrier F., Cau-Pottier E., Boutigny H., Roussel-Delvallez M., Jourdain M., Chopin C. (2000). Effects of dental plaque antiseptic decontamination on bacterial colonization and nosocomial infections in critically ill patients. Intensive Care Med..

[12] Beraldo C.C., Andrade D.d. (2008). Oral hygiene with chlorhexidine in preventing pneumonia associated with mechanical ventilation. J. Bras. Pneumol..

[13] Terezakis E., Needleman I., Kumar N., Moles D., Agudo E. (2011). The impact of hospitalization on oral health: A systematic review. J. Clin. Periodontol..

[14] Cecon F., Ferreira L.E.N., Rosa R.T., Gursky L.C., e Carvalho A.d.P., Samaranayake L.P., Rosa E.A.R. (2010). Time-related increase of staphylococci, enterobacteriaceae and yeasts in the oral cavities of comatose patients. J. Microbiol. Immunol. Infect..

[15] Binkley C., Furr L.A., Carrico R., McCurren C. (2004). Survey of oral care practices in US intensive care units. Am. J. Infect. Control.

[16] Holmes S. (1991). The oral complications of specific anticancer therapy. Int. J. Nurs. Stud..

[17] Ganz F.D., Fink N.F., Raanan O., Asher M., Bruttin M., Nun M.B., Benbinishty J. (2009). ICU nurses’ oral-care practices and the current best evidence. J. Nurs. Scholarsh..

[18] Eilers J., Berger A.M., Petersen M.C. (1988). Development, testing, and application of the oral assessment guide. Oncol. Nurs. Forum.

[19] Allen Furr L., Binkley C.J., McCurren C., Carrico R. (2004). Factors affecting quality of oral care in intensive care units. J. Adv. Nurs..

[20] Saddki N., Mohamad Sani F.E., Tin-Oo M.M. (2017). Oral care for intubated patients: A survey of intensive care unit nurses. Nurs. Crit. Care.

[21] Lin Y.S., Chang J.C., Chang T.H., Lou M.F. (2011). Critical care nurses’ knowledge, attitudes and practices of oral care for patients with oral endotracheal intubation: A questionnaire survey. J. Clin. Nurs..

[22] Jahani S., Poursangbor T. (2019). Survey of knowledge, attitude and performance of Intensive Care Unit nurses regarding oral care of patients under mechanical ventilation in educational hospitals of Ahvaz, 2017. J. Adv. Pharm. Educ. Res..

[23] Jun M.-K., Ku J.-K., Kim I.-h., Park S.-Y., Hong J., Kim J.-Y., Lee J.-K. (2021). Hospital dentistry for intensive care unit patients: A comprehensive review. J. Clin. Med..

[24] Adib-Hajbaghery M., Ansari A., Azizi-Fini I. (2013). Intensive care nurses’ opinions and practice for oral care of mechanically ventilated patients. Indian J. Crit. Care Med..

[25] Jordan A., Badovinac A., Špalj S., Par M., Šlaj M., Plančak D. (2014). Factors influencing intensive care nurses’ knowledge and attitudes regarding ventilator-associated pneumonia and oral care practice in intubated patients in Croatia. Am. J. Infect. control.

[26] Labeau S., Vandijck D., Rello J., Adam S., Rosa A., Wenisch C., Bäckman C., Agbaht K., Csomos A., Seha M. (2008). Evidence-based guidelines for the prevention of ventilator-associated pneumonia: Results of a knowledge test among European intensive care nurses. J. Hosp. Infect..

[27] Sole M.L., Byers J.F., Ludy J.E., Zhang Y., Banta C.M., Brummel K. (2003). A multisite survey of suctioning techniques and airway management practices. Am. J. Criti. Care.

[28] Cason C.L., Tyner T., Saunders S., Broome L. (2007). Nurses’ implementation of guidelines for ventilator-associated pneumonia from the Centers for Disease Control and Prevention. Am. J. Criti. Care.

